# Changes in the Salivary Glands in Diabetes

**Published:** 1899-10

**Authors:** 


### SELECTIONS.

### Changes in the Salivary Glands in Diabetes.

Dr. H. F. Harris (*Boston Med. and Surg. Journal,* May 18th) had his attention first directed to a possible relation between morbid conditions of the salivary glands and pancreas by the following case in which diabetes followed mumps. The patient was a farmer, aged forty-two, who had had no illness until an attack of mumps three years before. About a month after recovery he noticed that he was passing more urine than formerly, and since then the increase had slowly but steadily progressed and the other symptoms of diabetes appeared. He was much emaciated and passed in the twenty-four hours 6,440 cubic centimeters of urine, containing 4.6 percent of glucose. His subsequent history is unknown. The writer found in literature several references to the fact that diabetes sometimes follows mumps. He also ascertained that by the removal of all the salivary glands in a dog slight transient glycosuria could be produced—a conclusion which had been previously established by Reale (Congress of Berlin, 1899). Furthermore, the latter experimenter produced glycosuria in this way in dogs when he had failed to do so by removing the pancreas. Since the publication of Schmackpfeffer's thesis in 1817 on diseases of the pancreas the possibility of pancreatitis arising from mumps has been admitted, but there is some doubt as to the nature of the case to which he referred. Dr. Harris could not find another case of pancreatitis due to mumps. But when it is remembered that sublingual and submaxillary adenitis, orchitis, prostatitis, ovaritis, mastitis, nephritis and thyroïditis have been observed, it seems probable that a gland so closely resembling the parotid as does the pancreas would, at times, be involved. If so, as in the parotid and testicle, atrophy might ensue.

That functional alterations in the salivary glands occur in diabetes has long been known, but the condition has received little attention from clinicians and pathologists. The following case shows that profound changes sometimes occur in the salivary glands in every way resembling those found in the pancreas. A man, aged sixty-three, died from diabetes complicated with pulmonary tuberculosis and gangrene of the foot. The body was much emaciated. The pancreas was found to be very small, weighing only thirty-five grammes. It was hard and nodular and on section showed numerous fibrous bands. There was evidently atrophy secondary to old interstitial pancreatitis. All the salivary glands were small. Microscopically both the pancreas and salivary glands showed identical changes. There was marked thickening of the trabeculæ which bind the lobules together. In some parts all the acini of a lobule were destroyed and replaced by newly-formed fibrous tissue. Microscopical examination of the other organs showed them to be normal.—*Lancet.*

